# Supplementary material for: Japanese encephalitis virus neuropenetrance is driven by mast cell chymase
Source: Nat Commun. 2019 Feb 11;10:706. doi: 10.1038/s41467-019-08641-z (PMC6370868; doi:10.1038/s41467-019-08641-z)
Supplement: Supplementary file 1 — Supplementary Information [file 41467_2019_8641_MOESM1_ESM.pdf]

SUPPLEMENTARY INFORMATION

Japanese encephalitis virus neuropenetrance is driven by mast cell chymase

Justin T. Hsieh, Abhay P.S. Rathore, Gayathri Soundarajan, Ashley L. St. John

Supplementary Figures

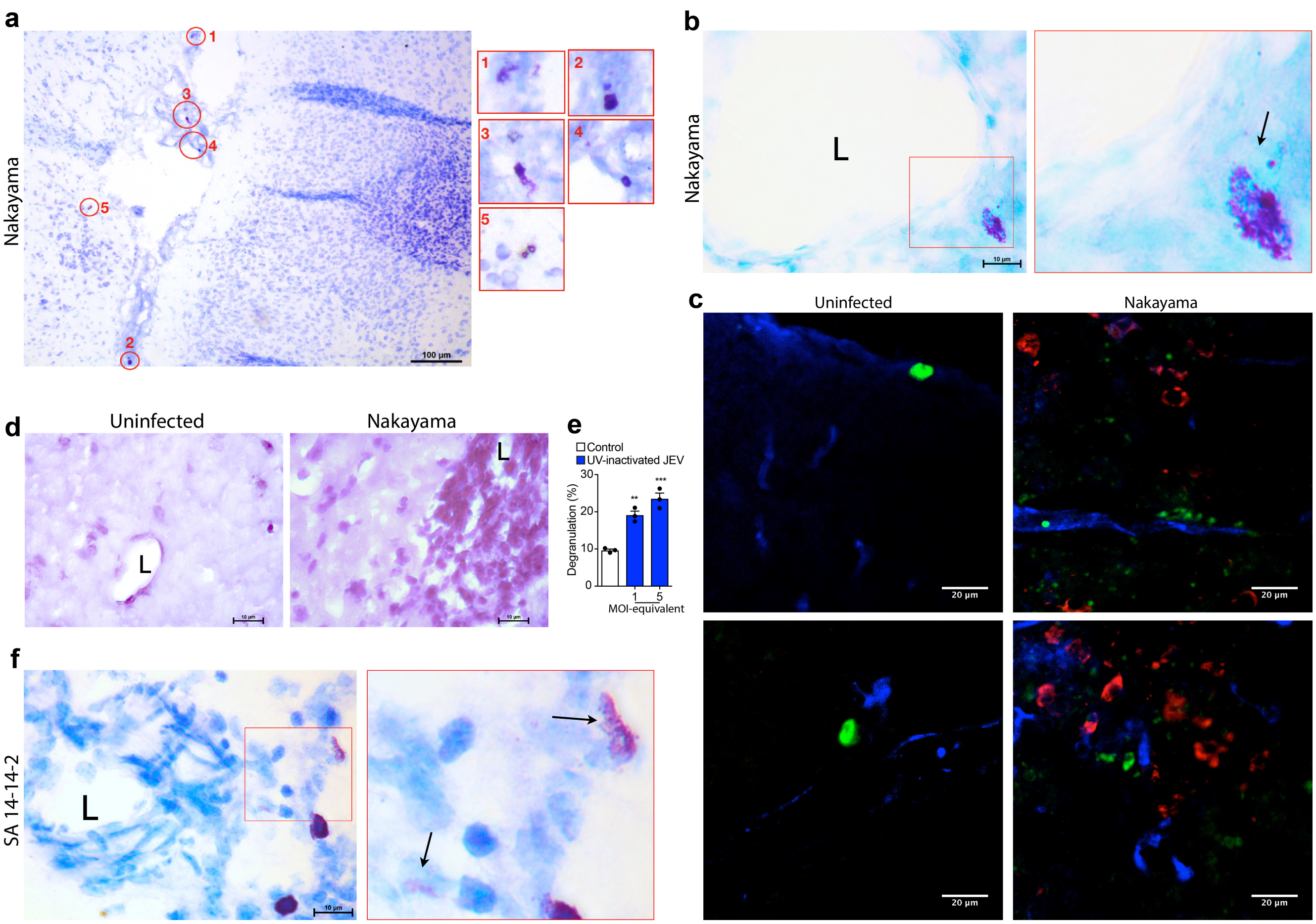

Supplementary Figure 1.
JEV induces MC activation, vascular compromise, and leukocyte infiltration into the brain.
(**a**) WT mice were infected *i.p.* with 2x10^7 plaque forming units (PFUs) of Nakayama JEV. Low-magnification image showing multiple degranulated MCs in the brain 5 days after Nakayama JEV infection. (**b**) JEV-activated MCs were noted near brain blood vessels, as assessed by toluidine blue staining. Boxed areas in the left image are magnified in the right image. An arrow points to the degranulated MC in this field, L= lumen. (**c**) Image of MC degranulation near endothelial cells (blue; CD-31) of the brain near areas of JEV infection (red; JEV NS3 protein), 5 days after *i.p.* Nakayama JEV infection. MC granules are stained using a probe for heparin (green). Vehicle-treated control brains showed well-granulated MCs. (**d**) H&E staining of JEV-infected mice brain showed vascular compromise and leukocyte infiltration 5 days after *i.p,* infection with Nakayama JEV (right image), compared to vehicle treated controls (left image), L= lumen. (**e**) UV inactivated SA14-14-2 JEV activates mouse BMMCs, measured by β-hexosaminidase assay. Statistics was performed using 1 way ANOVA with means compared to uninfected control and error bars represent the SEM. (**f**) *i.p.* injection of SA 14-14-2 JEV (4x10^7 PFU) also induced MC degranulation near brain blood vessels, as shown by toluidine blue staining. Arrows point to the degranulated MCs, L= lumen. All images are representative of n=3 mice.

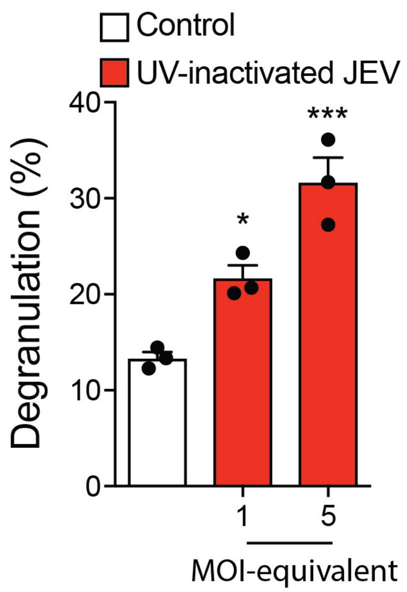

Supplementary Figure 2.
UV-inactivated JEV induces human MC degranulation.
Human ROSA cells exposed to UV-inactivated SA14-14-2 JEV degranulate in a concentration-dependent manner, as assessed by by β-hexosaminidase assay, analyzed using 1 way ANOVA with means compared to uninfected control. Error bars represent the SEM.

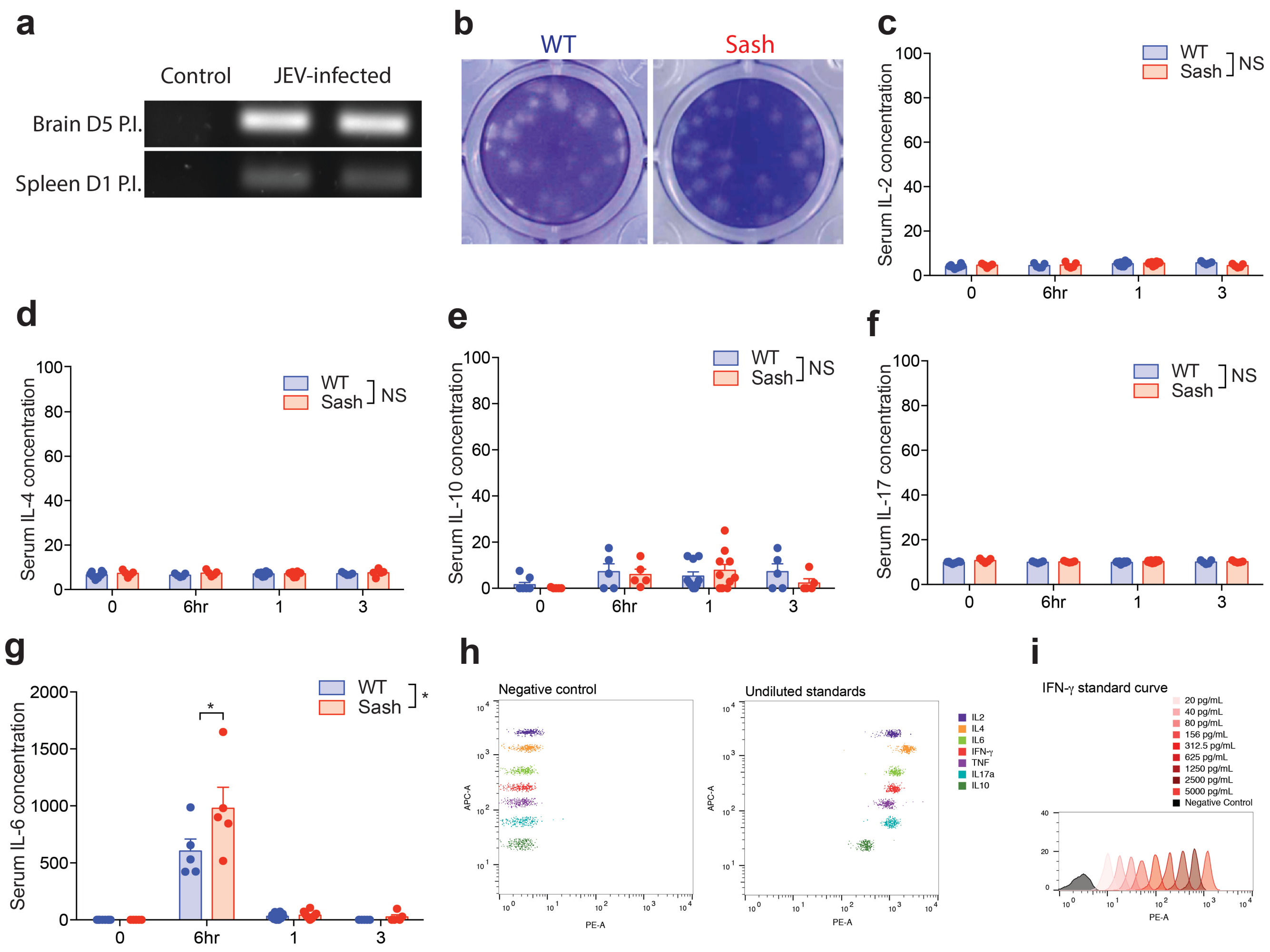

Supplementary Figure 3.

Similar levels of peripheral JEV infection and serum cytokines in WT and MC-deficient mice.

(**a**) PCR products showing detection of the JEV negative-strand in the brain at day 5 and spleen at day 1 post i.p. infection of mice confirming JEV replication *in vivo*. Uninfected tissues were used as controls. Representative images from n=5 animals are shown. P.I.= post infection. (**b**) Plaque assay demonstrating similar levels of infectious virus in the serum of WT and Sash mice 1 day after *i.p.* infection with SA-14-14-2 JEV. Representative image of serum plaque assay from n=5. Serum of JEV-infected WT and Sash mice had similar levels of multiple cytokines including (**c**) IL-2, (**d**) IL-4, (**e**) IL-10, and (**f**) IL-17. (**g**) Serum of Sash mice had higher levels of IL-6 as compared to WT mice at 6h post-infection and similar levels thereafter. * denotes $P < 0.05$. Error bars represent the SEM. Panels **c-g** were analyzed by 2-way ANOVA with Bonferroni's post-test, n=5 per group, representative of 3 independent experiments. (**h**) Representative flow cytometry plots demonstrating the positive and negative controls for the cytokine array assay and (**i**) a histogram presentation of a representative standard curve for the cytokine IFN-γ are provided.

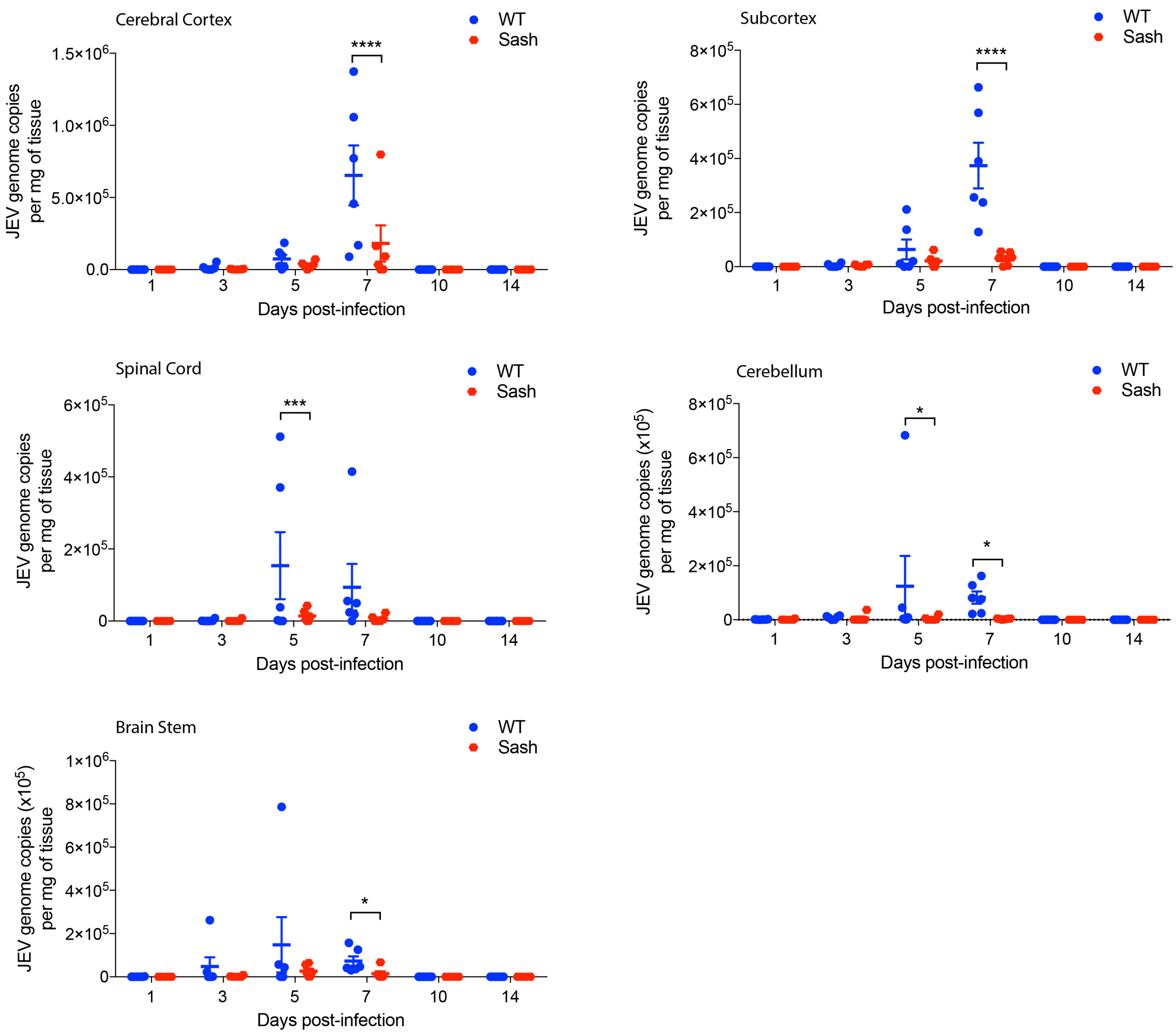

Supplementary Figure 4.
JEV burden in the brains of WT and Sash mice.
A dot-plot alternative representation of the data provided in Figure 2g. Error bars represent the SEM.

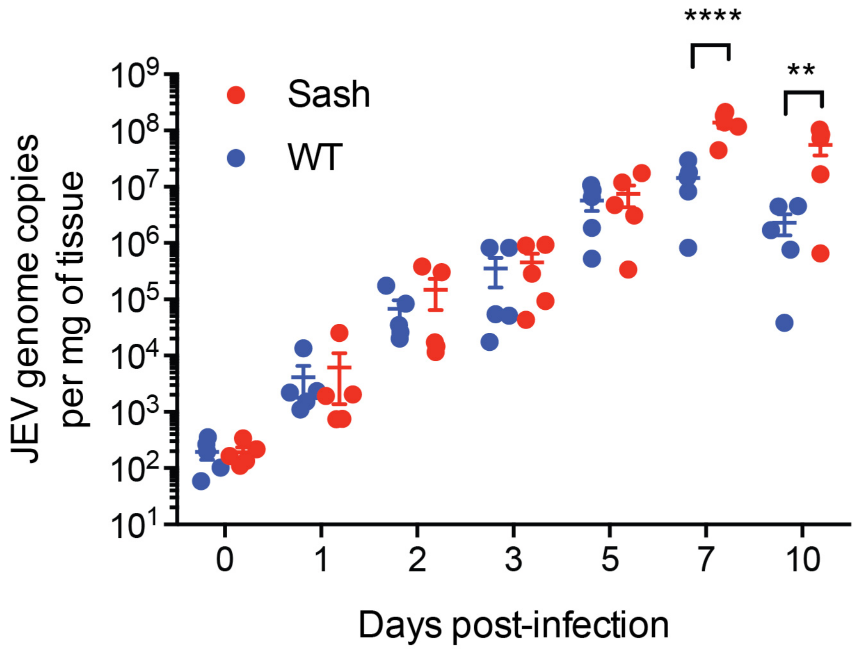

Supplementary Figure 5.

Kinetics of JEV infection after intracerebralventricular injection (i.c.v.) of virus in WT and MC-deficient mice.

JEV genome copies in the brain were detected by real time qPCR in WT and Sash after *i.c.v.* infection with SA 14-14-2 JEV ($1x10^4$ PFU). From days 1-5, no significant differences in viral burden were observed but burden was higher in Sash mice on days 7 and 10 post-infection. These results indicate defects in viral clearance in Sash mice when the brain is the site of inoculation. For all groups n=5 and data were analyzed by two way-ANOVA with Holm-Sidak's multiple comparison test to obtain *p*-values. Error bars represent the SEM. Data are compiled from two independent experiments. ** denotes $P < 0.01$ and *** denotes $P < 0.001$.

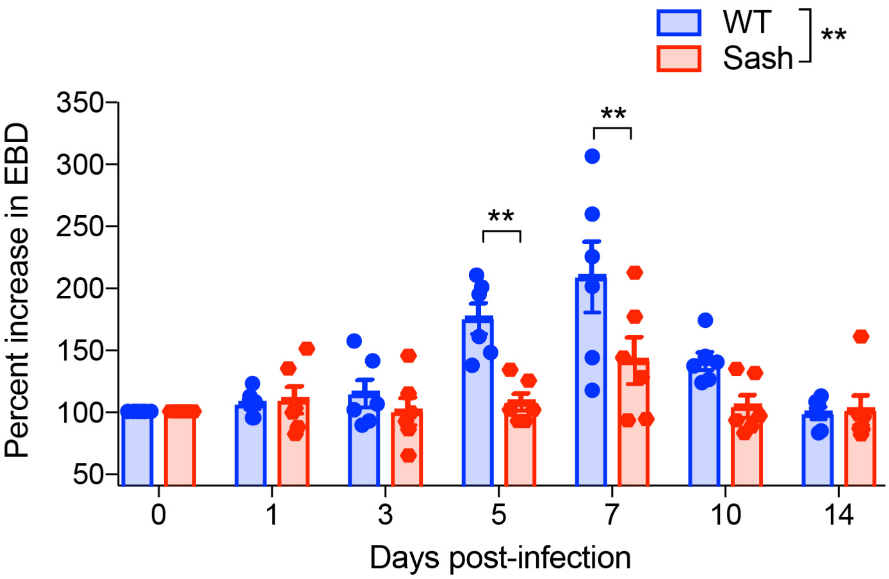

Supplementary Figure 6.
Peripheral JEV infection leads to increased BBB leakiness.
Alternative representation of Figure 3a showing a dot plot and mean±SEM. Graph depicts a time course of SA 14-14-2 JEV-induced BBB breakdown, detected by EBD leakage into the brain from 1 to 14 days after infection. EBD concentration was normalized to levels detected in vehicle treated controls. ** denotes *P <0.01* as analyzed by two-way ANOVA with Bonferroni's post-test; n=6. Error bars represent the SEM and data are representative of 3 independent experiments.

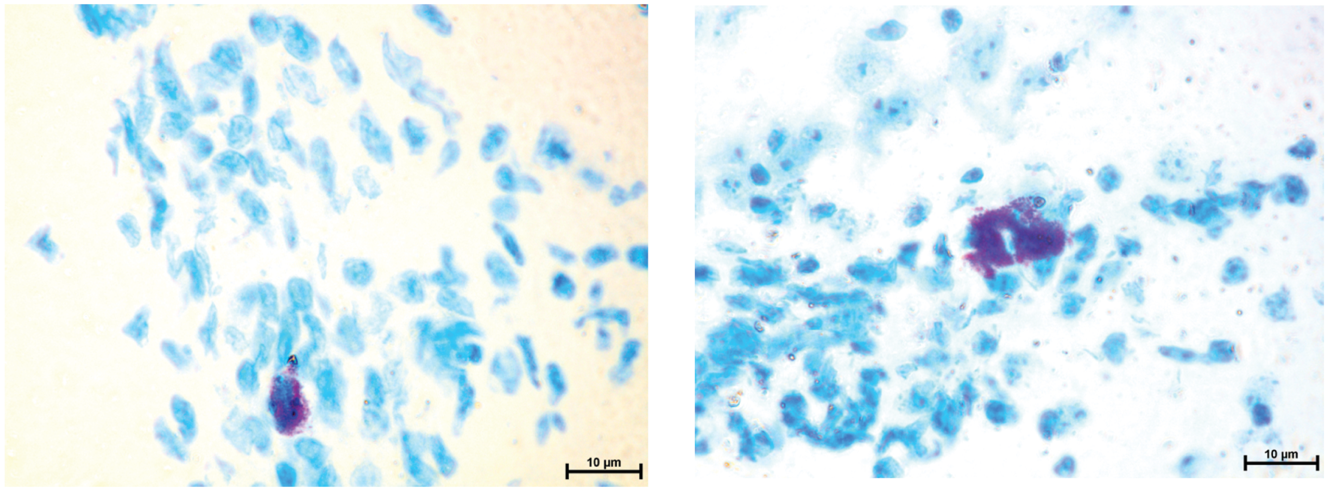

Supplementary Figure 7.
***I.c.v* injection of BMMCs leads to a low level of CNS MC-reconstitution.** Toluidine blue stained brain sections from tissues harvested 16 weeks after reconstitution of Sash mice by *i.c.v* reconstitution of $1x10^6$ BMMCs. Limited MC engraftment was viewed in the brain.

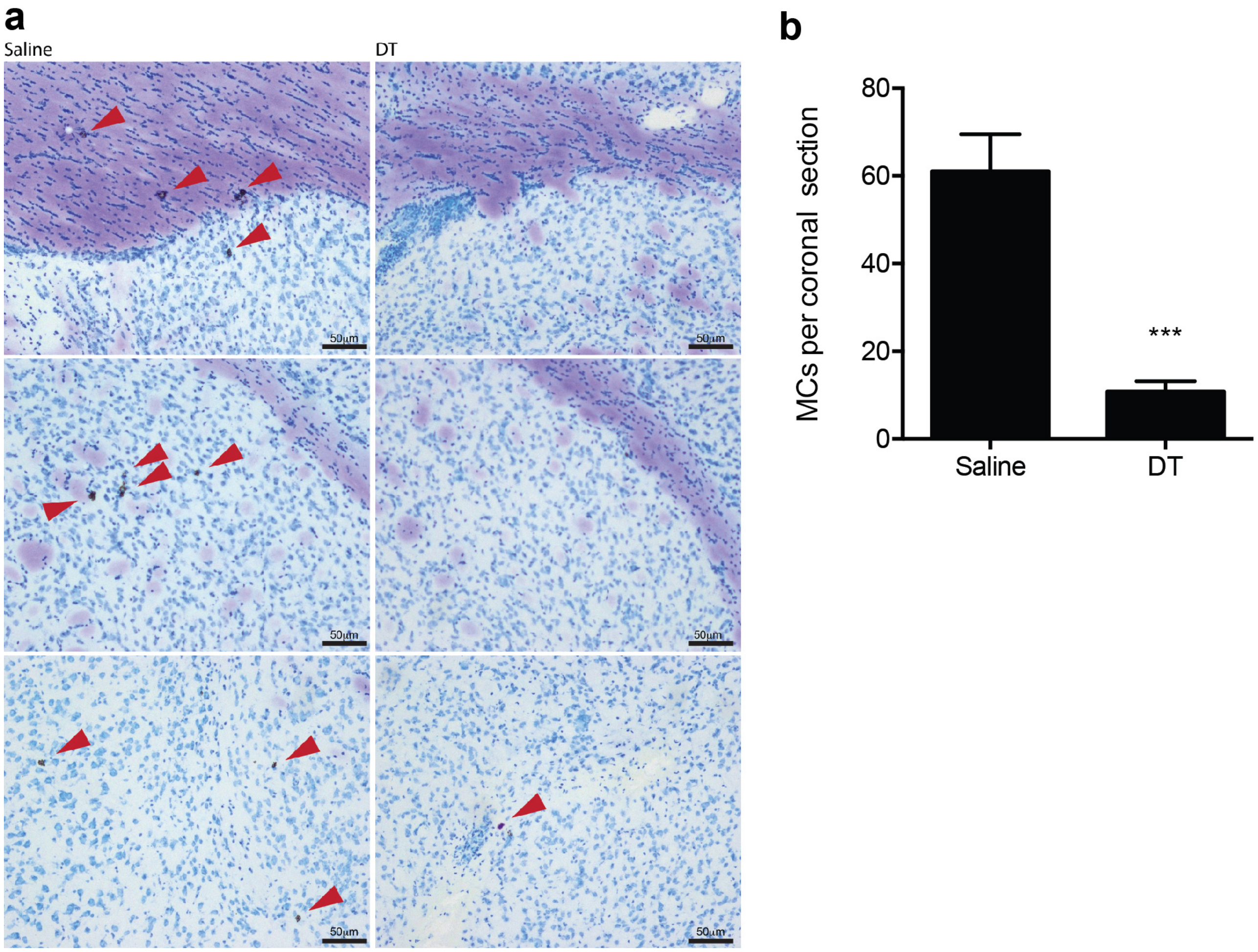

Supplementary Figure 8.

Injection of diphtheria toxin *i.c.v* into *Mcpt5-Cre* i-DTR$^{fl/fl}$ mice depletes ~80% of brain MCs.

(**a**) Representative images of brain sections showing reduced MCs in diphtheria toxin (DT)- injected mice and PBS-injected *Mcpt5-cre* i-DTR$^{fl/fl}$ mice. Red arrows point to metachromatic MCs. (**b**) Quantification of MC numbers in representative sections showing ~80% depletion of MCs by diphtheria toxin as compared to PBS injection. *** denotes $P < 0.001$ as analyzed by Student's unpaired *t*-test from representative sections derived from n=4 mice. Error bars represent the SEM.

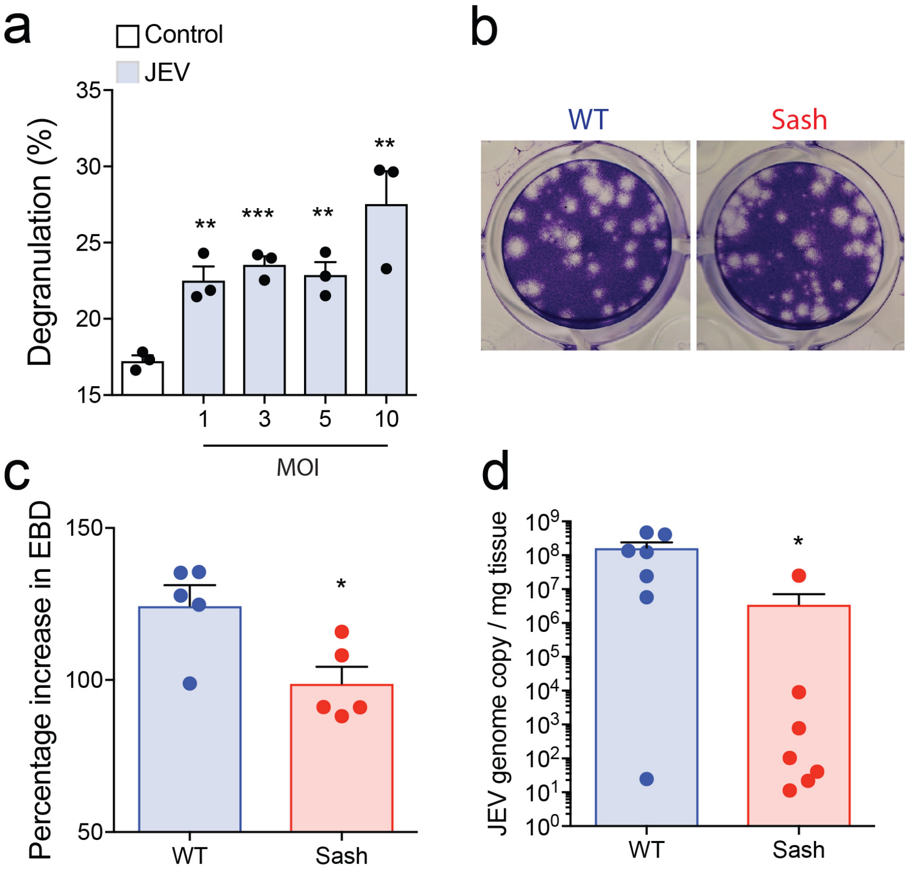

Supplementary Figure 9.
MCs promote BBB leakage and Nakayama JEV penetration into the brain.
(**a**) Nakayama JEV activates human ROSA cells, as assessed by β-hexosaminidase assay. * denotes *P <0.05*, ** denotes *P <0.01*, *** denotes *P <0.001* as analyzed by one-way ANOVA with Bonferroni's post-test. Graph is representative of 3 independent experiments. (**b**) Plaque assay demonstrating similar levels of infectious virus in the serum of WT and Sash mice at day 1 after *i.p.* infection with Nakayama JEV; representative plaque assay images are provided from n=5 mice. (**c**) JEV induces increased EBD leakage into the brains of WT mice compared to Sash mice, 3 days post-infection with JEV Nakayama by *i.p.* injection of $2x10^7$ PFU (n=5). (**d**) Increased virus infection was detected in the brains of WT mice compared to Sash mice 7 days post-Nakayama infection, as assessed by real time qPCR (n=8). For panels **c-d**, * denotes *P <0.05,* as analyzed by Student's unpaired *t*-test and data are representative of 3 independent experiments. Error bars represent the SEM.

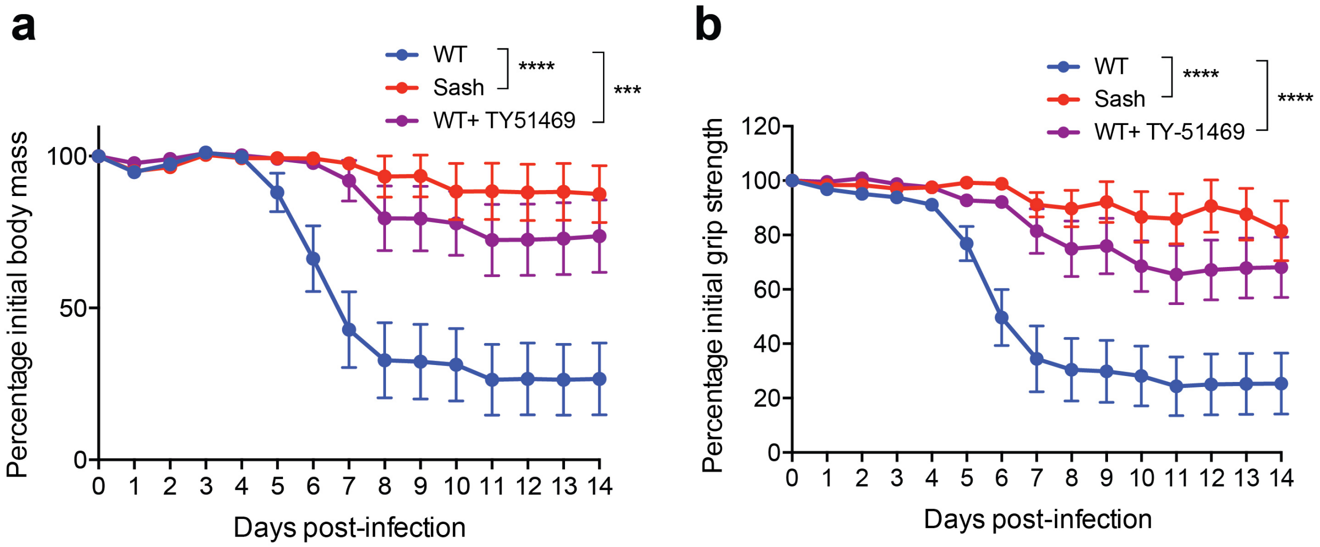

Supplementary Figure 10.
MCs contribute to morbidity in JEV-infected mice, which is reversed by chymase inhibition.
Body mass and grip strength of mice were measured daily and presented as a percentage of their pre-infection (day 0) body weight and grip strength. WT mice showed (**a**) increased loss in body mass and (**b**) worsened neurologic deficits after Nakayama JEV infection compared to Sash mice. *** denotes *P <0.001*, **** *denotes P <0.0001* as analyzed by two-way ANOVA; n=15 from three independent experimental replicates involving n=5 per group. Error bars represent the SEM. These figures correspond to the data in Figures 4d-e and 6a-b, which showed one experimental replicate involving n=5 per group, respectively.

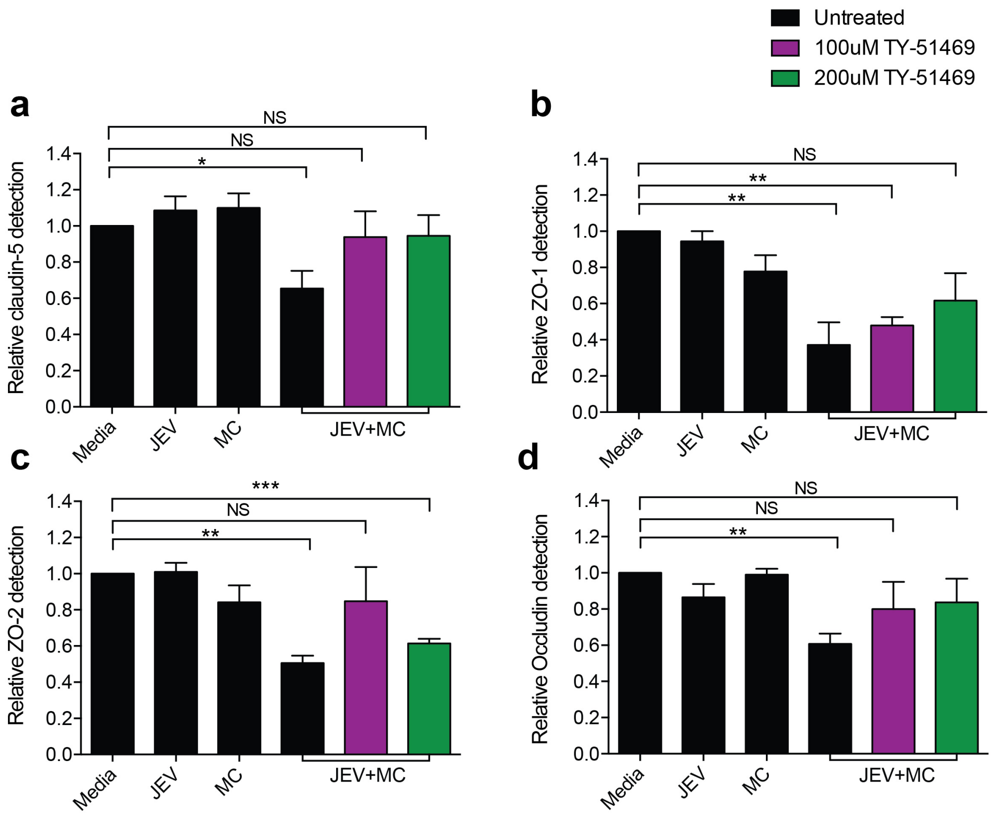

Supplementary Figure 11.
Quantification of Western blot bands by densitometry of bEND.3 tight junction proteins.
Quantification of Western blot bands from Figure 5e showed that bEND.3 cells exposed to JEV-activated supernatant demonstrated reduced (**a**) claudin-5, (**b**) ZO-1, (**c**) ZO-2, and (**d**) occludin levels when compared to media, JEV, and resting MC-stimulated controls. Chymase inhibition with TY-51469 at one or both doses (100uM and 200uM) reduced the breakdown of these TJ proteins. Protein levels were normalized to GAPDH. *denotes $P < 0.05$, **denotes $P < 0.01$, and ***denotes $P < 0.001$, as analyzed by individual groups to controls from 3 independent experiments by Student's unpaired *t*-test. Error bars represent the SEM.

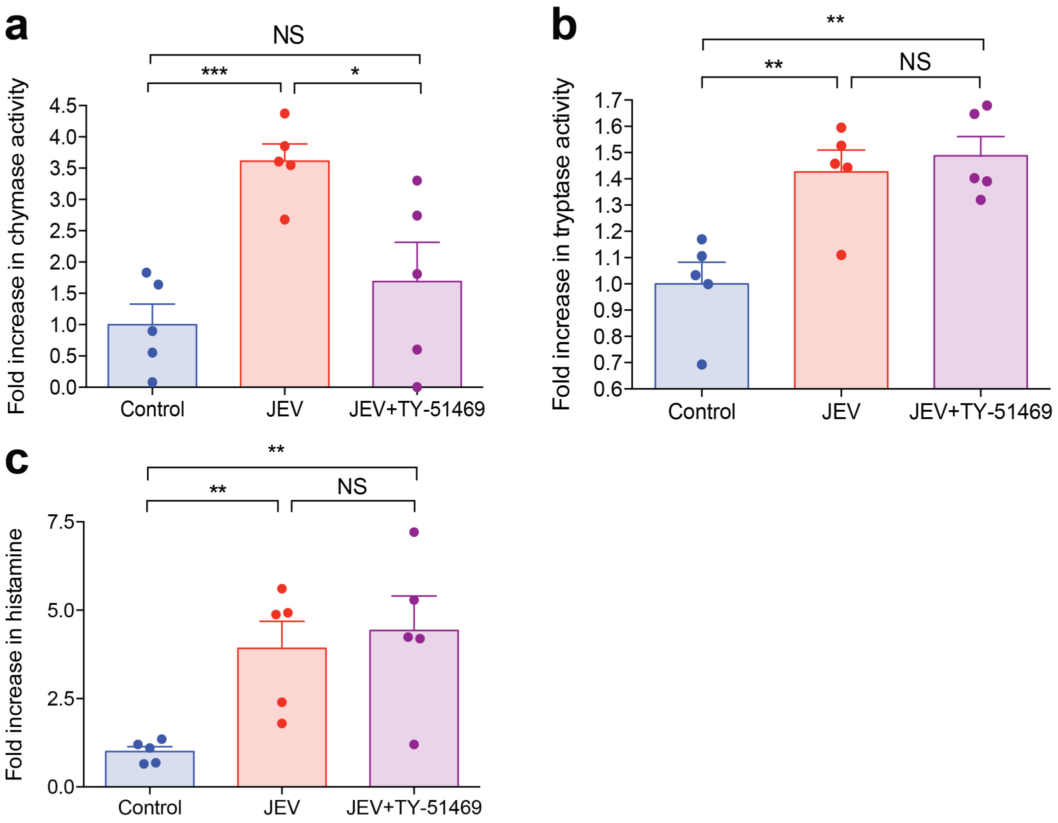

Supplementary Figure 12.
Chymase inhibitor TY-51469 treatment specifically inhibits chymase activity but not tryptase activity or histamine concentration in WT mice.
(**a**) Serum of WT mice had increased chymase activity as compared to Sash mice. Inhibition of chymase with TY-51469 led to reduced chymase activity to level of Sash mice. Serum of WT mice showed increased (**b**) tryptase activity and (**c**) histamine concentration as compared to Sash mice. *denotes $P<0.05$, **denotes $P<0.01$, ***denotes $P<0.001$, as analyzed by individual Student's unpaired *t*-tests. N=5 mice, representative of 3 independent experiments. Error bars represent the SEM. Treatment with TY-51469 did not alter the activity of tryptase or concentration of histamine.

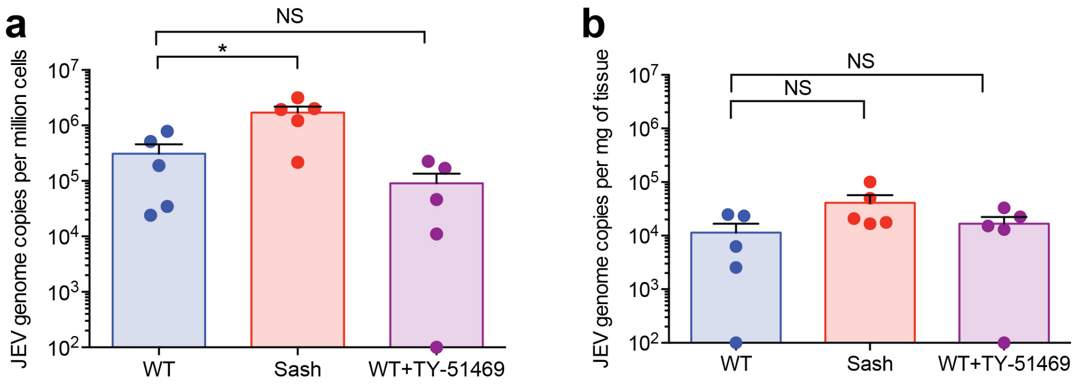

Supplementary Figure 13.
Inhibition of MC-specific protease chymase does not affect JEV clearance from the periphery.
Virus genome copies in (**a**) peritoneal cells and (**b**) spleen were detected by real time qPCR at 1 day after *i.p.* infection with Nakayma JEV (2x10^7 PFU). Chymase inhibitor TY-51469-treated WT mice showed no difference in clearance of JEV from the peritoneal cells or the spleen as compared to vehicle-treated WT mice. Sash mice showed significantly increased genome copies in peritoneal cells but not the spleen compared to WT and WT+TY-51469-treated mice. * denotes $P<0.05$; n=5, representative of 3 independent experiments. Error bars represent the SEM.

Supplementary Tables

Supplementary Table 1: PCR Primers used for JEV detection

<u>JEV 5' UTR</u>		
Amplification	Forward:	5'-AAAAACCAGGAGGGCCCGG-3' (nucleotides 80-104)
	Reverse:	5'-TTTACAGCATTAGCCCCGACCAA-3' (nucleotides 135-154)
<u>Negative-strand of JEV NS5</u>		
cDNA synthesis		5'-TACTCCGACGGTGTGGTCTA-3' (nucleotides 10224-10243)
Amplification	Forward:	5'-GCCGGGTGGGACACTAGAAT-3' (nucleotides 9288-9307)
	Reverse:	5'-TGGACAGCGATGTTCGTGAA-3' (nucleotides 9519-9538)

Supplementary Methods

Chymase and tryptase activity assays and histamine ELISA

Enzymatic activity of chymase and selectivity of the chymase inhibitor were assessed using the *Chymase Assay Kit* (Sigma-Aldrich, St. Louis, MO; #CS1140), *Mast cell Degranulation Tryptase Assay Kit* (Merk-Millipore, Burlington, MA; #IMM001) and histamine ELISA kits (Abcam, Cambridge, UK; #ab213975). Serum was collected from mice and used for the assays according to manufacturers' instructions. The chymase and tryptase activity and histamine level within the serum were normalized to the serum activity levels of vehicle-injected control mice.
