# Supplementary material for: Japanese encephalitis virus neuropenetrance is driven by mast cell chymase
Source: Nat Commun. 2019 Feb 11;10:706. doi: 10.1038/s41467-019-08641-z (PMC6370868; doi:10.1038/s41467-019-08641-z)
Supplement: Supplementary file 3 — Description of Additional Supplementary Files [file 41467_2019_8641_MOESM3_ESM.docx]

**Description of Additional Supplementary Files**

**File Name**: Supplementary Data 1

**Description**: Un-cut and annotated western blot and gel images.
