# Supplementary material for: Japanese encephalitis virus neuropenetrance is driven by mast cell chymase
Source: Nat Commun. 2019 Feb 11;10:706. doi: 10.1038/s41467-019-08641-z (PMC6370868; doi:10.1038/s41467-019-08641-z)

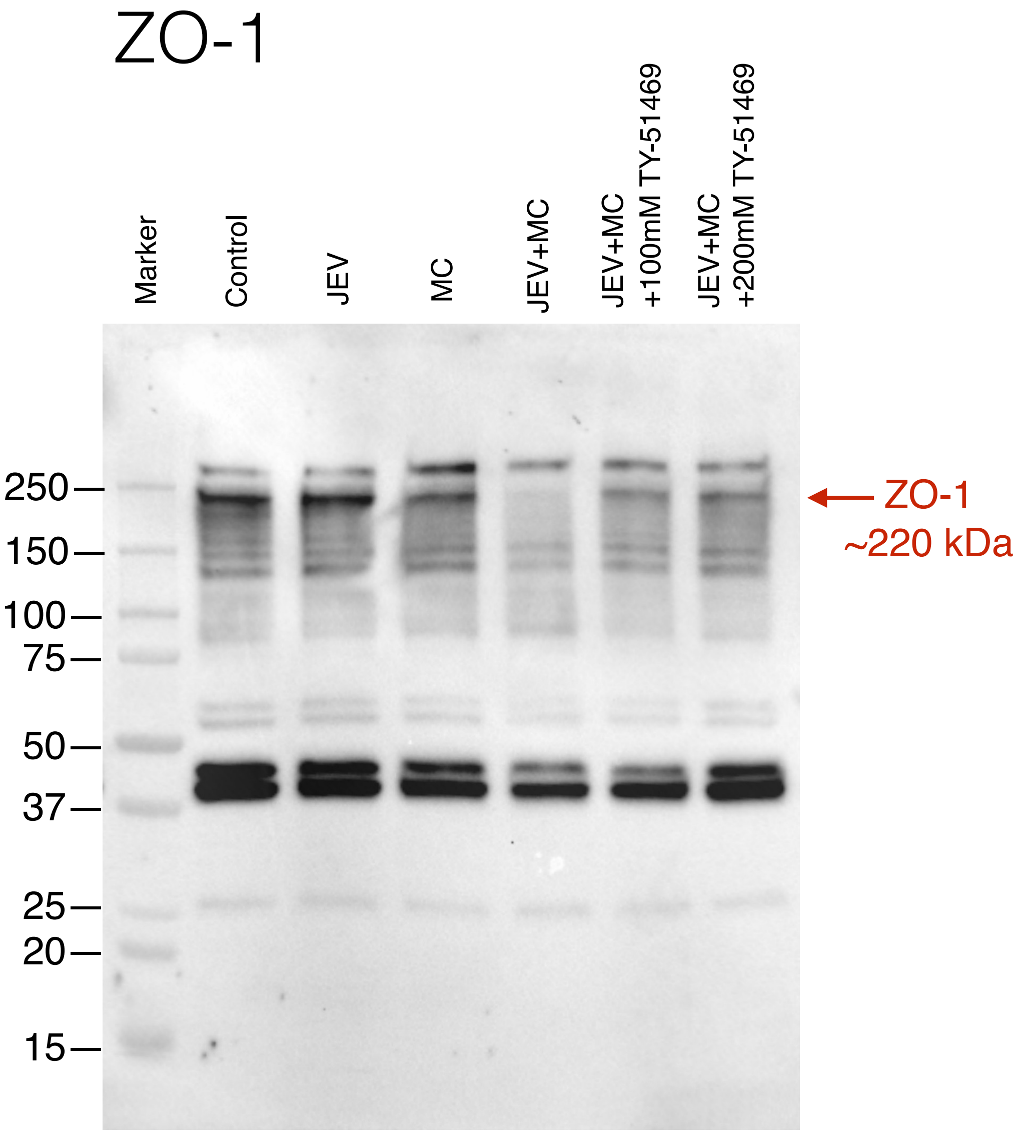
ZO-1
Marker
Control
JEV
MC
JEV+MC
JEV+MC +100mM TY-51469
JEV+MC +200mM TY-51469
250
150
100
75
50
37
25
20
15
ZO-1 ~220 kDa

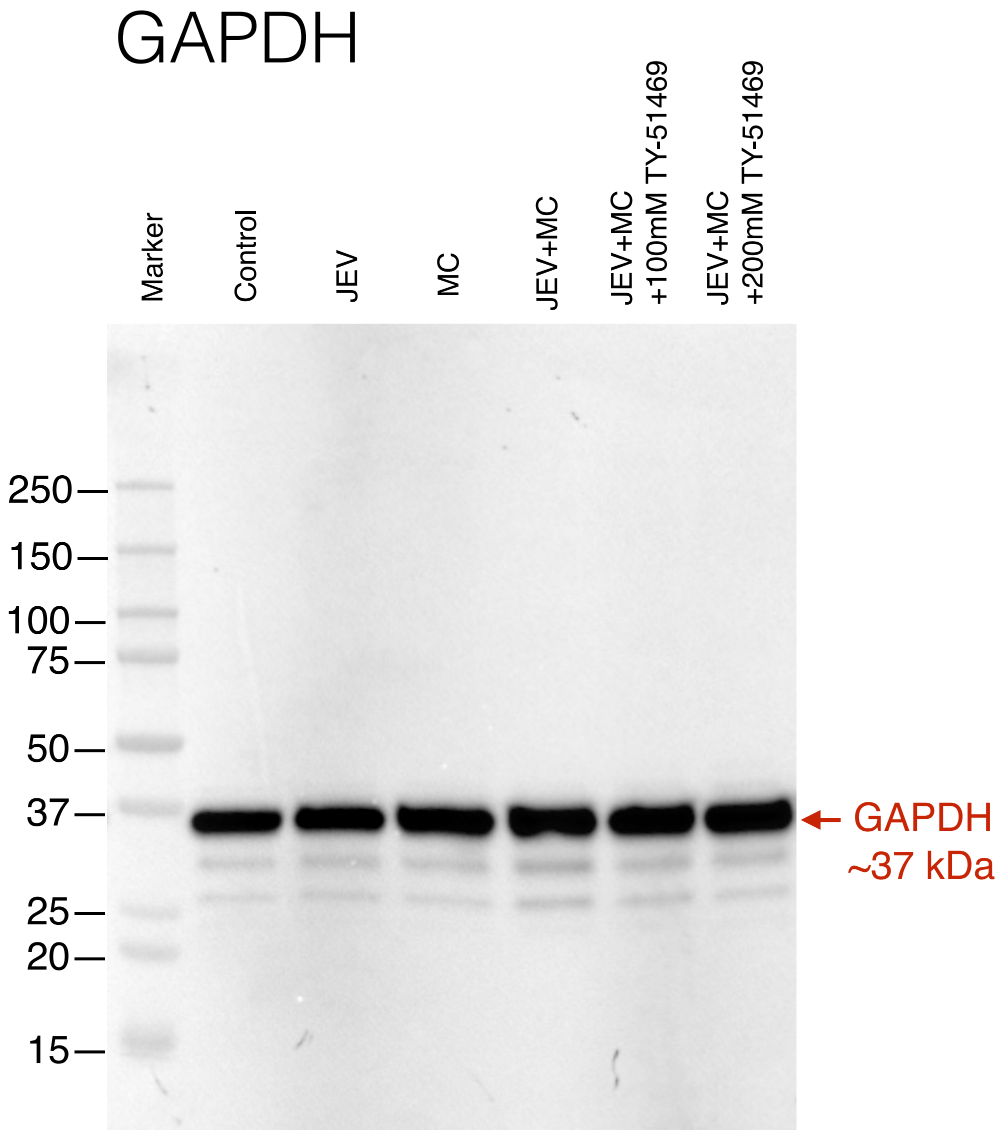
GAPDH
Marker
Control
JEV
MC
JEV+MC
JEV+MC +100mM TY-51469
JEV+MC +200mM TY-51469
250
150
100
75
50
37
25
20
15
GAPDH ~37 kDa

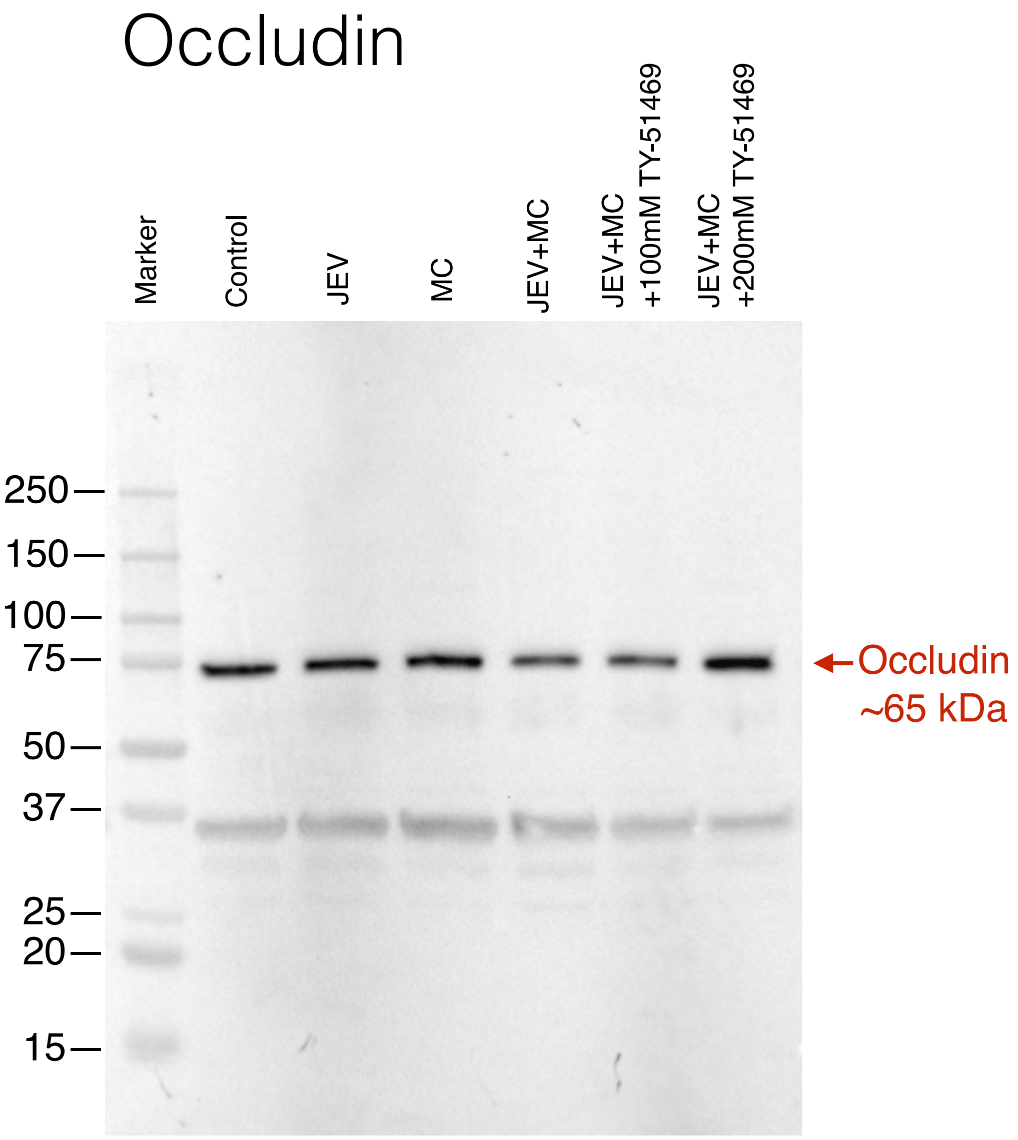

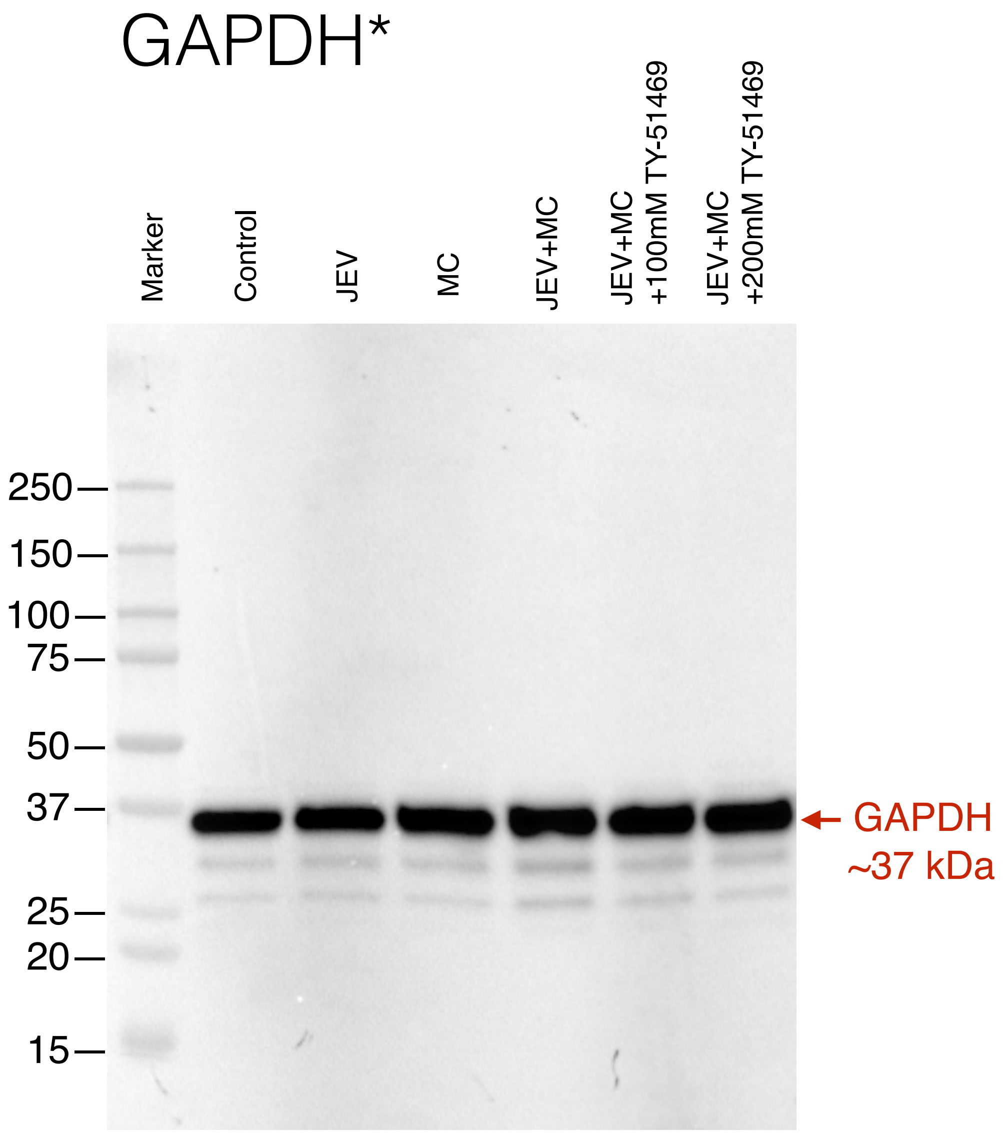

*Note that the same blot was probed for ZO-1, Occludin and GAPDH

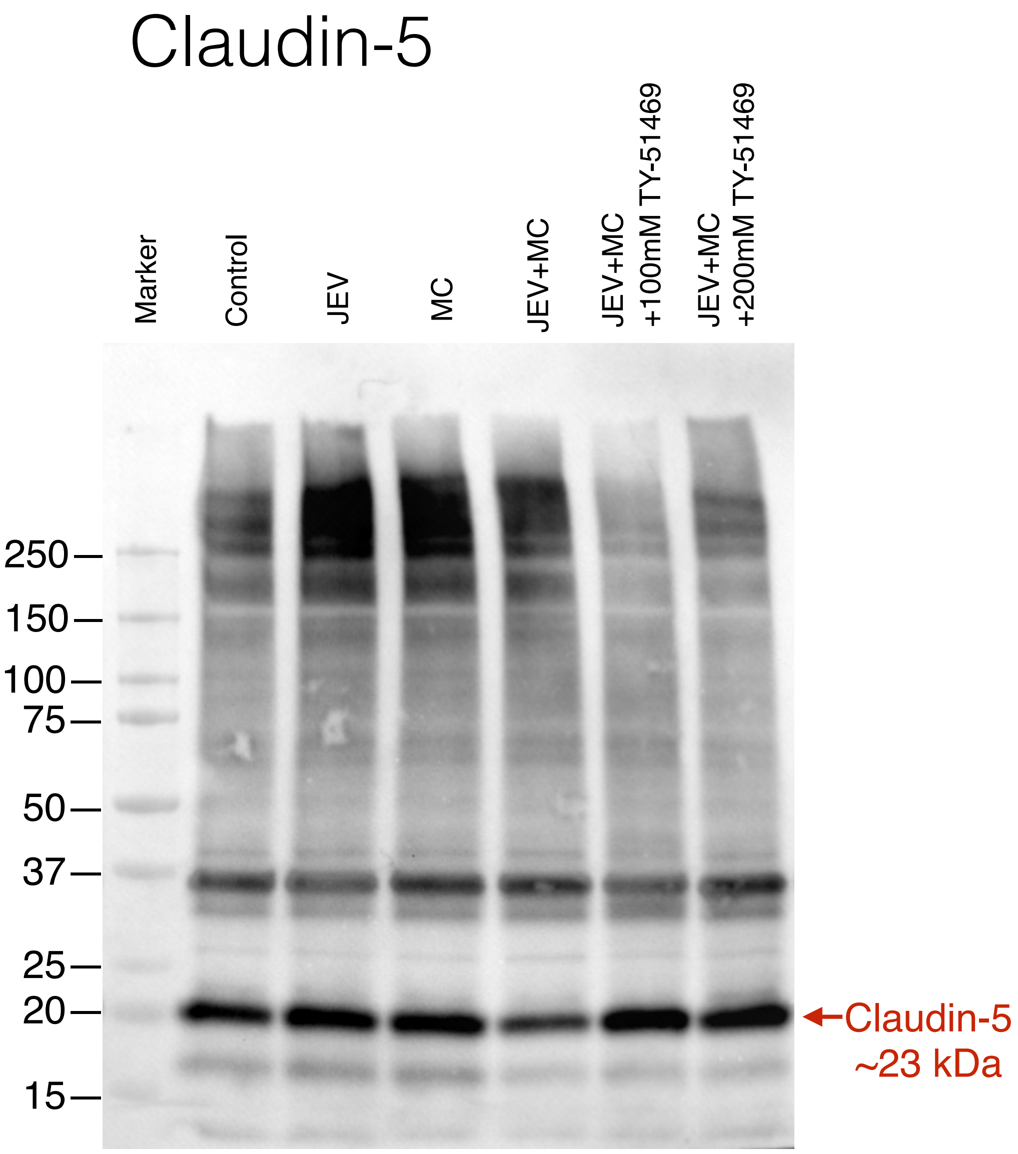
Claudin-5
Marker
Control
JEV
MC
JEV+MC
JEV+MC +100mM TY-51469
JEV+MC +200mM TY-51469
250
150
100
75
50
37
25
20
15
Claudin-5 ~23 kDa

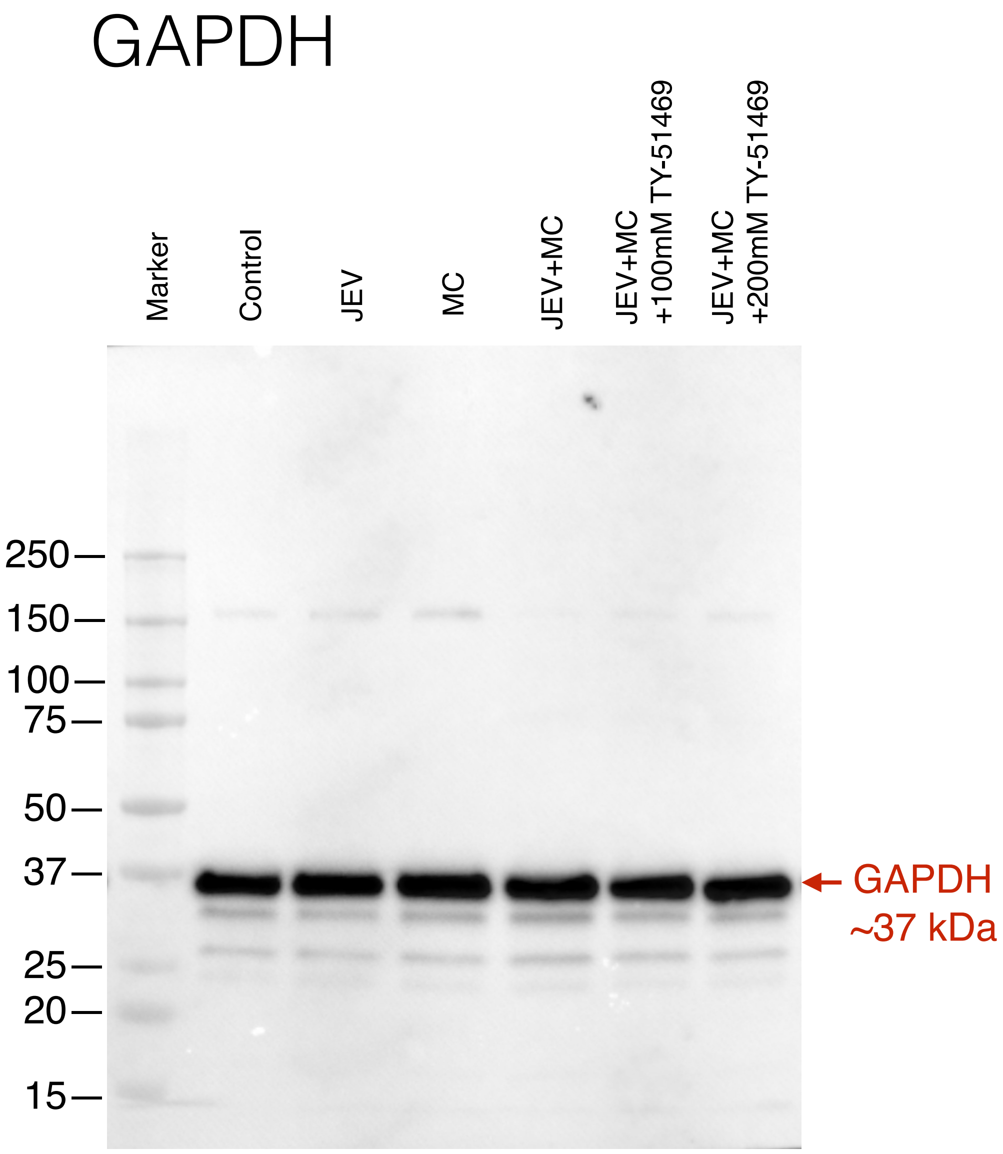
GAPDH
Marker
Control
JEV
MC
JEV+MC
JEV+MC +100mM TY-51469
JEV+MC +200mM TY-51469
250
150
100
75
50
37
25
20
15
GAPDH ~37 kDa

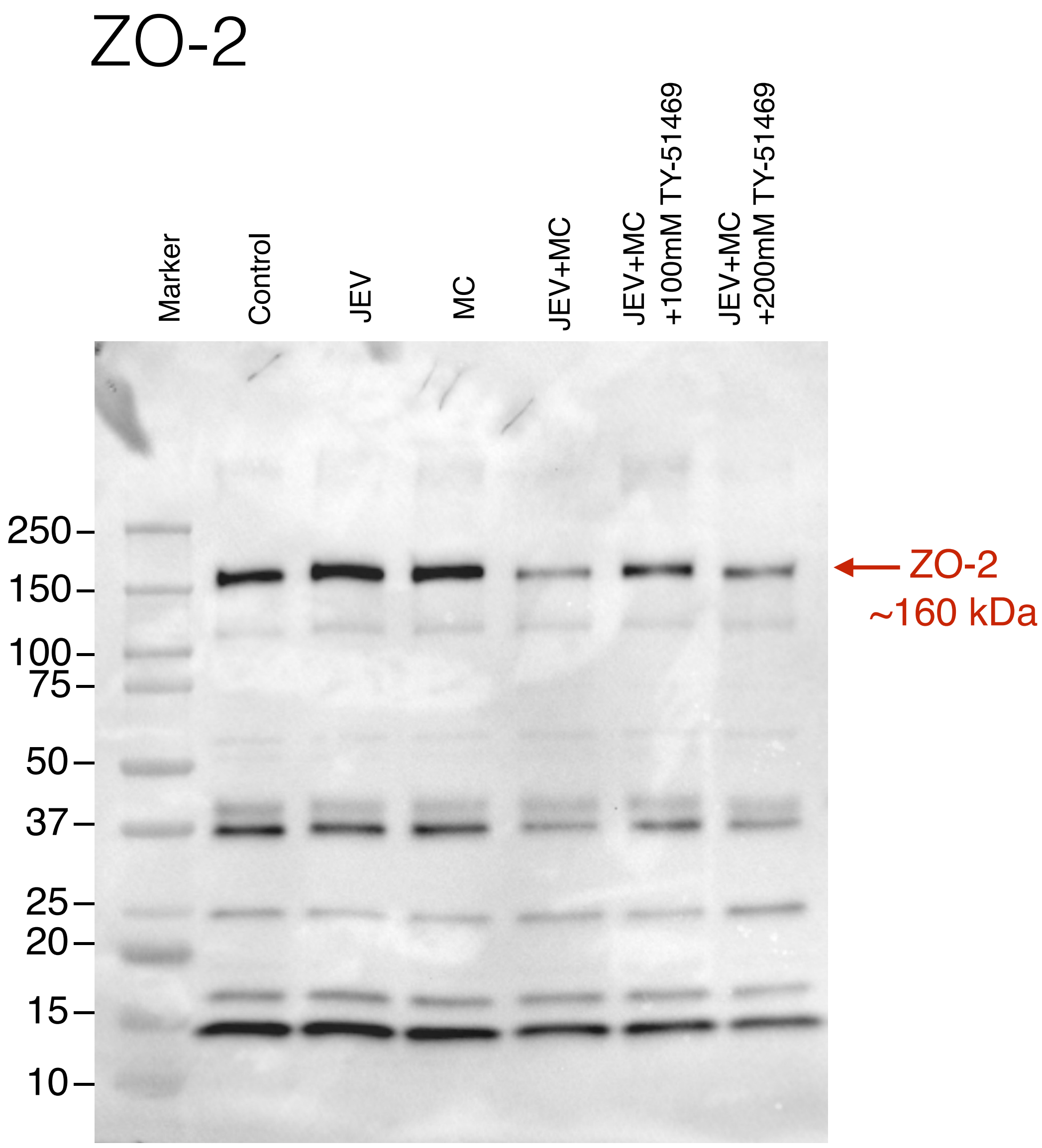
ZO-2
Marker
Control
JEV
MC
JEV+MC
JEV+MC +100mM TY-51469
JEV+MC +200mM TY-51469
250
150
100
75
50
37
25
20
15
10
ZO-2
~160 kDa

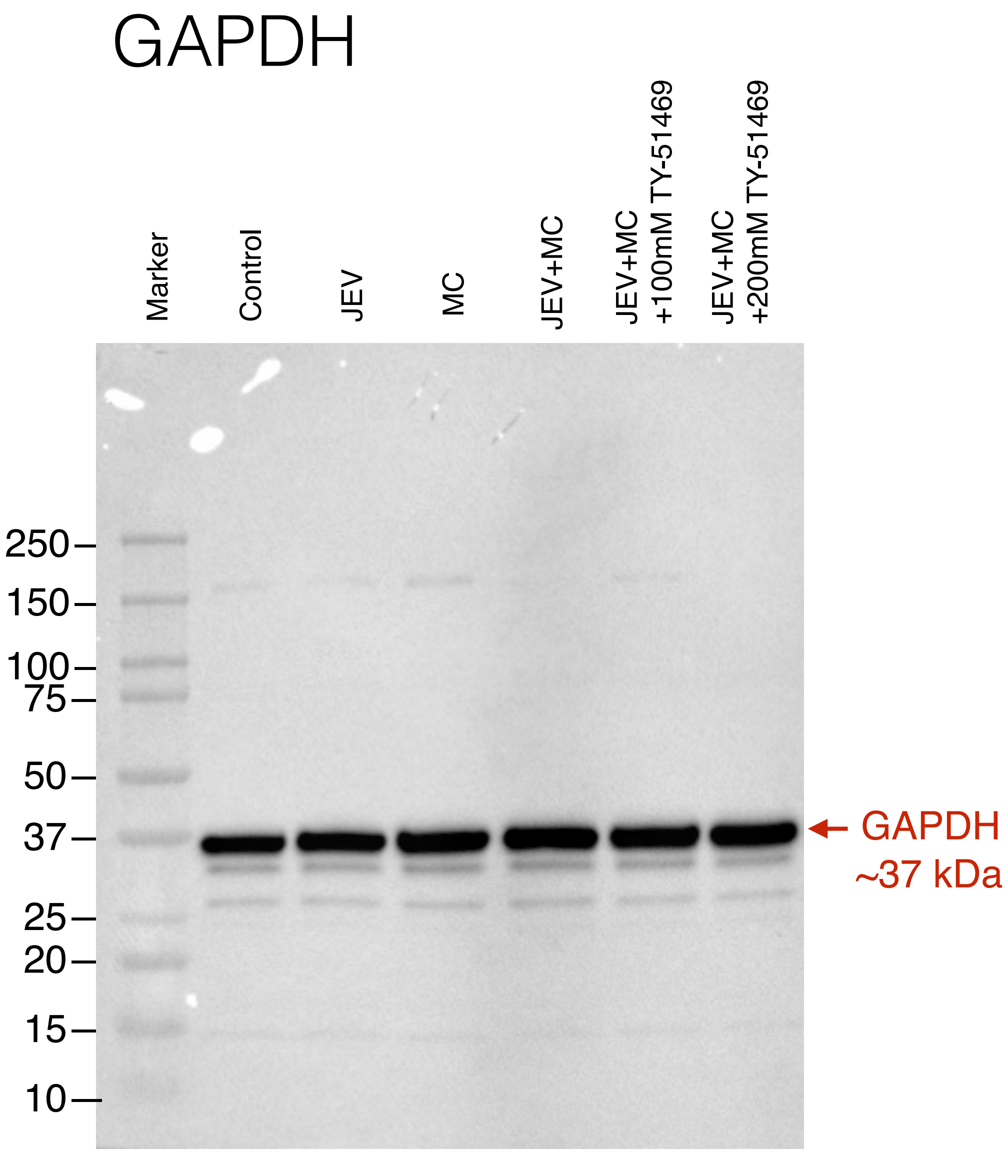
GAPDH
Marker
Control
JEV
MC
JEV+MC
JEV+MC +100mM TY-51469
JEV+MC +200mM TY-51469
250
150
100
75
50
37
25
20
15
10
GAPDH
~37 kDa

JEV negative-strand PCR

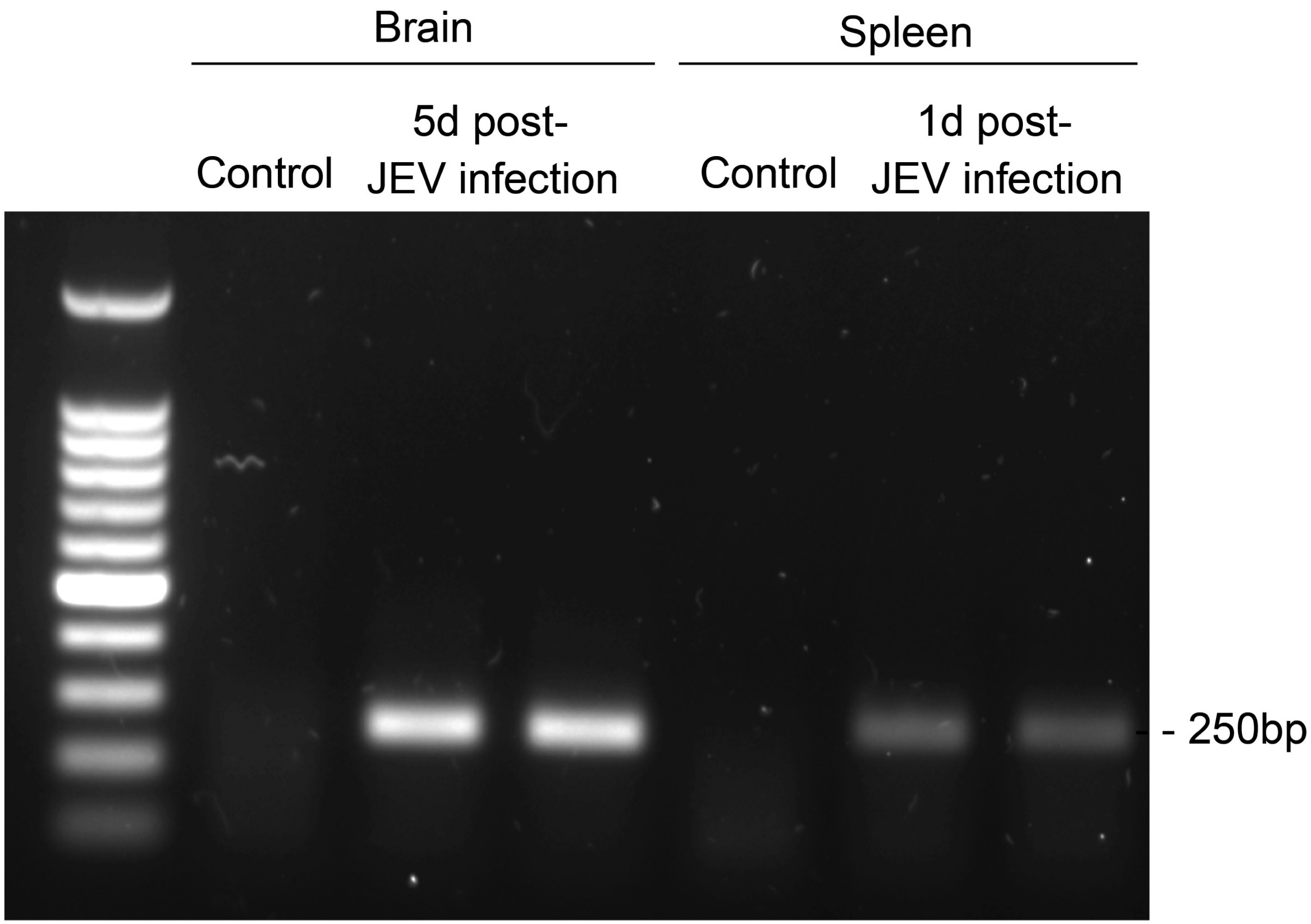

Supplement: Supplementary file 4 — Supplementary Data 1 [file 41467_2019_8641_MOESM4_ESM.pdf]
