# Supplementary material for: Japanese encephalitis virus neuropenetrance is driven by mast cell chymase
Source: Nat Commun. 2019 Feb 11;10:706. doi: 10.1038/s41467-019-08641-z (PMC6370868; doi:10.1038/s41467-019-08641-z)
Supplement: Supplementary file 5 — Reporting Summary [file 41467_2019_8641_MOESM5_ESM.pdf]

nature research

Corresponding author(s): Ashley St. John

Last updated by author(s): Jan 15, 2019

Reporting Summary

Nature Research wishes to improve the reproducibility of the work that we publish. This form provides structure for consistency and transparency in reporting. For further information on Nature Research policies, see Authors & Referees and the Editorial Policy Checklist.

Statistics

For all statistical analyses, confirm that the following items are present in the figure legend, table legend, main text, or Methods section.

n/a	Confirmed	
☐	☒	The exact sample size (*n*) for each experimental group/condition, given as a discrete number and unit of measurement
☐	☒	A statement on whether measurements were taken from distinct samples or whether the same sample was measured repeatedly
☐	☒	The statistical test(s) used AND whether they are one- or two-sided<br>*Only common tests should be described solely by name; describe more complex techniques in the Methods section.*
☒	☐	A description of all covariates tested
☒	☐	A description of any assumptions or corrections, such as tests of normality and adjustment for multiple comparisons
☐	☒	A full description of the statistical parameters including central tendency (e.g. means) or other basic estimates (e.g. regression coefficient) AND variation (e.g. standard deviation) or associated estimates of uncertainty (e.g. confidence intervals)
☒	☐	For null hypothesis testing, the test statistic (e.g. *F*, *t*, *r*) with confidence intervals, effect sizes, degrees of freedom and *P* value noted<br>*Give P values as exact values whenever suitable.*
☒	☐	For Bayesian analysis, information on the choice of priors and Markov chain Monte Carlo settings
☒	☐	For hierarchical and complex designs, identification of the appropriate level for tests and full reporting of outcomes
☒	☐	Estimates of effect sizes (e.g. Cohen's *d*, Pearson's *r*), indicating how they were calculated

Our web collection on statistics for biologists contains articles on many of the points above.

Software and code

Policy information about availability of computer code

Data collection	Not applicable
Data analysis	Not applicable

For manuscripts utilizing custom algorithms or software that are central to the research but not yet described in published literature, software must be made available to editors/reviewers. We strongly encourage code deposition in a community repository (e.g. GitHub). See the Nature Research guidelines for submitting code & software for further information.

Data

Policy information about availability of data

All manuscripts must include a data availability statement. This statement should provide the following information, where applicable:

- Accession codes, unique identifiers, or web links for publicly available datasets
- A list of figures that have associated raw data
- A description of any restrictions on data availability

Data availability statement has been included in the manuscript.

Field-specific reporting

Please select the one below that is the best fit for your research. If you are not sure, read the appropriate sections before making your selection.

☒ Life sciences ☐ Behavioural & social sciences ☐ Ecological, evolutionary & environmental sciences

For a reference copy of the document with all sections, see nature.com/documents/nr-reporting-summary-flat.pdf

Life sciences study design

All studies must disclose on these points even when the disclosure is negative.

Sample size	Sample size calculations were performed when designing the study.
Data exclusions	Data were not excluded.
Replication	All experiments were repeated 2-3 independent times.
Randomization	Not applicable, because in-bred animals were used for all experiments.
Blinding	Blinding was not performed for the safety of the researcher working with infected samples.

Reporting for specific materials, systems and methods

We require information from authors about some types of materials, experimental systems and methods used in many studies. Here, indicate whether each material, system or method listed is relevant to your study. If you are not sure if a list item applies to your research, read the appropriate section before selecting a response.

Materials & experimental systems

n/a	Involved in the study	
☐	☒	Antibodies
☐	☒	Eukaryotic cell lines
☒	☐	Palaeontology
☐	☒	Animals and other organisms
☒	☐	Human research participants
☒	☐	Clinical data

Methods

n/a	Involved in the study	
☒	☐	ChIP-seq
☐	☒	Flow cytometry
☒	☐	MRI-based neuroimaging

Antibodies

Antibodies used	Antibodies were purchased for the applications of immunostaining and western blotting. All catalogue numbers are included in the manuscript.
Validation	Validated antibodies were purchased and used.

Eukaryotic cell lines

Policy information about cell lines

Cell line source(s)	All cell lines except the ROSA human mast cell line, which was obtained from Prof. Michel Arock, were obtained from ATCC.
Authentication	None of the cell lines were independently authenticated. They were purchased from sources providing certificates of analysis with the exception of the ROSA cell line.
Mycoplasma contamination	Cell lines were tested for mycoplasma contamination and found to be negative.
Commonly misidentified lines (See ICLAC register)	None.

Animals and other organisms

Policy information about studies involving animals; ARRIVE guidelines recommended for reporting animal research

Laboratory animals	C57Bl/6 mice were used for this study.
Wild animals	None
Field-collected samples	None
Ethics oversight	Animal protocols were approved by the SingHealth IACUC.

Note that full information on the approval of the study protocol must also be provided in the manuscript.

Flow Cytometry

Plots

Confirm that:

☒ The axis labels state the marker and fluorochrome used (e.g. CD4-FITC).

☒ The axis scales are clearly visible. Include numbers along axes only for bottom left plot of group (a 'group' is an analysis of identical markers).

☒ All plots are contour plots with outliers or pseudocolor plots.

☐ A numerical value for number of cells or percentage (with statistics) is provided.

Methodology

Sample preparation	Flow cytometry was used to measure cytokines (not cells) using the mouse Th1/Th2/Th17 cytokine kit (BD Biosciences, Singapore; #560485). For this reason no cell percentages need to be reported and the sample preparation was specific to cytokine analysis.
Instrument	*Identify the instrument used for data collection, specifying make and model number.*
Software	Flowjo
Cell population abundance	Flow cytometry was used to measure cytokine levels by a specialized assay so cell populations are not reported.
Gating strategy	Flow cytometry data analysis strategy with representative plots is provided in Supplementary Figure 3h-i

☒ Tick this box to confirm that a figure exemplifying the gating strategy is provided in the Supplementary Information.
